# Health Behaviors and Health Status among Middle-Aged and Older Adults with Chronic Diseases in Taiwan

**DOI:** 10.3390/ijerph17197196

**Published:** 2020-10-01

**Authors:** Wei-Hua Tian, Joseph J. Tien

**Affiliations:** 1 Department of Economics, National Cheng Kung University, Tainan 701, Taiwan; 2 Department of Risk Management and Insurance, Tamkang University, New Taipei City 251, Taiwan; jilltien@mail.tku.edu.tw

**Keywords:** health behavior, health status, latent class analysis, middle-aged and older adults

## Abstract

Changes in lifestyle behaviors may effectively maintain or improve the health status of individuals with chronic diseases. However, such health behaviors adopted by individuals are unlikely to demonstrate similar patterns. This study analyzed the relationship between the heterogeneous latent classes of health behavior and health statuses among middle-aged and older adults with hypertension, diabetes, or hyperlipidemia in Taiwan. After selecting 2103 individuals from the 2005 and 2009 Taiwan National Health Interview Survey (NHIS), we first identified heterogeneous groups of health behaviors through latent class analysis (LCA). We further explored the relationship between each latent class of health behavior and health status through ordered logit regression. We identified the following five distinct health behavior classes: the all-controlled, exercise and relaxation, healthy diet and reduced smoking or drinking, healthy diet, and least-controlled classes. Regression results indicated that individuals in classes other than the all-controlled class all reported poor health statuses. We also found great magnitude of the coefficient estimates for individuals who reported their health status to be poor or very poor for the least-controlled class. Therefore, health authorities and medical providers may develop targeted policies and interventions that address multiple modifiable health behaviors in each distinct latent class of health behavior.

## 1. Introduction

Previous studies concerning unhealthy lifestyle behaviors, including an unhealthy diet, lack of sufficient daily physical activity, smoking, and drinking, have established that these behaviors contribute to poor health, mortality, and chronic disease [1,2,3,4,5,6,7,8,9]. Studies have also revealed that changes in lifestyle patterns through the adoption of health behaviors can effectively improve an individual’s health conditions or alleviate symptoms of chronic diseases. For example, reduced intake of sodium, regular exercise, no smoking, and limited drinking are all recommended methods for controlling chronic diseases [10,11,12]. Previous findings have indicated that changes in lifestyle patterns exhibited by healthy individuals or individuals who have chronic diseases can be grouped into clusters [13,14,15,16,17,18,19,20,21,22,23,24,25,26,27]. Individuals, therefore, can be classified into heterogeneous clusters of health behaviors, and these behaviors may have synergistic effects on health status [13,16]. For example, Bush et al. (2013) suggested that relatively fewer unhealthy behaviors were associated with less problematic physical and psychosocial health outcomes for Dutch adolescents [13]. Conry et al. (2011) highlighted that Irish adults with healthier patterns of behavior were associated with lower levels of psychological distress, higher levels of energy, better self-reported health status, and better quality of life [16]. Furthermore, according to the statistics from the World Health Organization, chronic diseases are responsible for approximately 70% of all deaths globally [28]. Therefore, this study analyzed the relationship between health behavior clusters and health status among middle-aged and older adults with chronic diseases in Taiwan. We proposed the hypothesis that relatively fewer healthy behavior clusters and self-reported health status are negatively related.

In particular, cardiovascular disease (CVD) has been identified as the leading cause of deaths globally [29], also ranking second among the ten major causes of death in Taiwan in 2016 [30]. The major risk factors contributing to CVD are hypertension, diabetes, and hyperlipidemia. According to the 2016 statistics from the Ministry of Health and Welfare in Taiwan, hypertensive disease was the main cause of death for people aged ≥40 years [30]. Individuals aged ≥40 years are responsible for approximately 77% of the total outpatient and inpatient expenses under Taiwan’s National Health Insurance program. However, in 2016, this age group accounted for only 58.6% of the total Taiwanese population [31]. The middle-aged and older populations are often affected by these chronic conditions [20], which are also associated with greater health care utilization and costs. Therefore, identifying risk factors and promoting health management through positive health behavior changes are critical. Considering that the proportion of older adults in Taiwan’s population is estimated to increase over the coming decade [32], a useful approach for controlling escalating health care costs involves maintaining and improving the health status of middle-aged and older adults with chronic diseases by promoting positive health behaviors in this demographic is important. Liu and Su (2017) revealed that health behaviors were robust predictors of healthy aging [33]. Therefore, an analysis of the relationship between health behaviors and health status among middle-aged and older adults (aged ≥40 years) with hypertension, diabetes, or hyperlipidemia in Taiwan warrants research attention.

Health is the most valuable aspect of our lives. Dever (1976) indicated that, in an epidemiological model, lifestyle was the most vital of four factors affecting health, with the others being human biology, environment, and the health care system [34]. Moreover, previous empirical evidence has emphasized that an unhealthy lifestyle contributes to poor health, mortality, and chronic diseases [1,2,3,4,5,6,7,8,9]. For example, McGinnis and Foege (1993) identified the three main factors that contribute to mortality in the United States, namely smoking, drinking, and an unhealthy diet [6]. Kenkel (1995) employed 1985 Health Interview Survey data to examine how health status is influenced by the “Alameda 7” indicators of a healthy lifestyle: diet, smoking, drinking, exercise behaviors, sleep, weight, and stress status. He reported that excessive weight, cigarette smoking, heavy drinking, excessive or insufficient sleep, and stress tend to exert negative effects on health, whereas exercise and moderate alcohol consumption have positive effects [5]. Particularly, an unhealthy diet and sedentary lifestyle are associated with obesity, cardiometabolic syndrome, type 2 diabetes, and mental health problems [35]. Unhealthy lifestyle behaviors are associated with poor health status; however, these behaviors are modifiable. Researchers have discussed that changes in lifestyle patterns or adoption of health behaviors may effectively improve the conditions of those with chronic diseases, such as hypertension and diabetes [10,11,12]. Therefore, positive changes in health behaviors by reducing the identified modifiable lifestyle risk factors could prevent specific chronic diseases and promote health, thereby confirming the importance of preventive medicine.

Preventive medicine prevents diseases and promotes health. The structured model of preventive medicine proposed by Leavell and Clark (1965) follows a three-level, five-stage strategy [36]. At the primary level, prevention focuses on health promotion to avert the onset of illness or injury before disease progression. The secondary level involves the identification of specific illnesses or conditions at an early stage for prompt interventions to prevent or limit disability. The tertiary level relates to the measures implemented to mitigate the symptoms of chronic diseases, permanent disability, and death. Studies have examined whether the early detection, diagnosis, and immediate treatment of diseases can reduce the risk of developing permanent disabilities and explored corresponding health service utilization and expenditure at the secondary level [37,38,39,40,41]. However, only a few studies have examined health strategies such as health behaviors and their effect on the health promotion of individuals with chronic diseases at the tertiary level [10,11,12,42]. Therefore, this study analyzed the relationship between health behavior clusters and health status among middle-aged and older adults with chronic diseases at the tertiary level. Although previous studies have often investigated the role played by demographic characteristics and socioeconomic background in predicting health behavior clusters in older adults with chronic diseases [20], the relationship between clusters of health behaviors and health status remains understudied. Therefore, this study certainly would shed light on preventive medicine research.

## 2. Materials and Methods

### 2.1. Data Resources and Study Sample

We obtained data from the 2005 and 2009 National Health Interview Survey (NHIS). The National Health Research Institute, the Health Promotion Administration, and the Ministry of Health and Welfare in Taiwan have been conducting the NHIS using face-to-face interviews and multistage stratified systematic sampling every 4 years since 2001. The NHIS provides nationwide detailed population information on individual characteristics, including age, gender, marital status, educational attainment, and income. Moreover, the survey provides detailed data on personal health conditions and health-related behaviors, such as lifestyle changes that improve or maintain the health status of individuals with chronic diseases. The protocol has been approved by Institutional Review Board (IRB) of National Cheng Kung University Hospital (IRB number: A-ER-102-364-t). We selected individuals aged ≥40 years with hypertension, diabetes, or hyperlipidemia and those who reported having drinking or smoking habits. The research sample comprised 2103 individuals from Taiwan. Among them, 62.62%, 23.54%, and 52.97% of the respondents had hypertension, diabetes, or hyperlipidemia, respectively. Moreover, 13.17% of the respondents had hypertension and diabetes, 22.73% had hypertension and hyperlipidemia, and 9.27% had diabetes and hyperlipidemia; 5.75% of respondents had all these conditions.

Table 1 presents the sample characteristics of the study population. Among the 2103 respondents, the self-reported health status of only 1.71% was deemed excellent compared with that of the previous year, whereas 8.04%, 51.69%, 32.19%, and 6.37% reported their health status compared with that of the previous year to be good, fair, poor, and very poor, respectively. In terms of age, 57.82% of the respondents were aged 40–59 years and lived with 3.15 people on average. Moreover, 79.17% of the respondents were male, 79.22% were married, 71.37% were of Taiwanese ethnicity, 41.32% had a senior high school diploma or above, and 38.09% lived in northern Taiwan. Regarding average monthly income, 75.32% of the respondents earned less than NT$40,000, and 18.78% earned between NT$40,000 and NT$79,999. Regarding respondents’ reported diseases, chronic diseases including cancer and heart, stroke, kidney, lung, or liver diseases covered 33.1% of the sample, and other chronic diseases including gout, arthritis, or osteoporosis covered 31.76%. Among the respondents, 49.74%, 20.26%, and 17.31% were taking medication for hypertension, diabetes, and hyperlipidemia, respectively.

### 2.2. Empirical Model

To explore health behavior clusters, we first identified heterogeneous groups of health behaviors among middle-aged and older adults with hypertension, diabetes, or hyperlipidemia in Taiwan using latent class analysis (LCA). By analyzing clusters of health behaviors rather than single behaviors, we aimed to highlight the importance of health behavior patterns among heterogeneous individuals. Second, we explored the relationship between each latent class of health behavior and health status using ordered logit regression.

#### 2.2.1. Latent Class Analysis

LCA is used to identify latent classes within a population on the basis of a set of individual responses to the discrete manifest variables (the observed indicators). LCA has been widely adopted in medical studies and health research. We used it in our study to create mutually exclusive groups of people for whom the within-group differences in the discrete manifest responses for health behaviors were minimal and between-group differences were maximized. LCA examines unobserved heterogeneity, mitigating the possibility of biased estimates due to heterogeneity in conventional regression. It is a person-centered approach used to reveal the smallest differences with respect to health behaviors among individuals within the same class. LCA constitutes a finite mixture model [43]. Theoretically, it assumes conditional independence, indicating that the latent class variable can explain the associations among the manifest variables. When the latent variable is known and has a constant value, the manifest variables are independent of each other under the LCA assumption. The expectation–maximization algorithm is normally employed for maximum likelihood estimation (MLE) in LCA. LCA is utilized to estimate the conditional probabilities of each health behavior indicator on the basis of MLE. In this study, we used a single-stage exploratory LCA. To identify latent classes, we modeled all manifest variables simultaneously and employed the Mplus 6.0 program (Mplus Office, Los Angeles, CA, USA) for estimation [44]. 

#### 2.2.2. Ordered Logit Model

We examined the relationship between each latent class of health behavior and health status using an ordered logit model. In this study, we defined the dependent variable (self-reported health status) according to the responses to the following survey question: “In general, compared with last year, how would you rate your own health at present—excellent, good, fair, poor, or very poor (from 1 to 5)?” We used the ordered logit model because self-reported health status requires an ordered response and linear regression is inappropriate when the dependent variable is categorical. The ordered logit regression was conducted using Stata 14 (StataCorp., College Station, TX, United States) [45].

The key independent variable was latent class classification based on health behavior clusters among middle-aged and older adults with hypertension, diabetes, or hyperlipidemia. On the basis of the responses to the survey question, “Are you currently controlling hypertension, diabetes, or hyperlipidemia through lifestyle changes?”, we constructed the latent classes of health behaviors on the basis of the following seven dichotomous indicators: weight control, reduction in smoking or drinking, regular exercise, healthy diet, relaxation or a stress-free lifestyle, meditation, and use of other methods to help control hypertension, diabetes, or hyperlipidemia. The baseline chronic disease statuses and health behaviors of the sample were cross-sectional. Therefore, we were unable to determine the exact health status and time at which individuals developed hypertension, diabetes, or hyperlipidemia and the timing and duration of health behavior changes. Notwithstanding the limitations, our study’s approach was appropriate for assessing whether lifestyle changes can benefit the health status of those with chronic conditions because the adopted survey questions investigated behavioral changes over time.

To classify the latent classes of health behaviors, we employed a series of model fit criteria, including Pearson’s chi-squared test, likelihood ratio (LR) chi-squared test, Akaike information criterion (AIC), and Bayesian or adjusted Bayesian information criterion (BIC/adjusted BIC). We conducted the Vuong–Lo–Mendell–Rubin likelihood ratio (LMR) test to compare the model fit between sequential classes. We also included other control variables, such as whether respondent has hypertension, diabetes, or hyperlipidemia drug treatment; their socioeconomic background; and other disease statuses. Socioeconomic background was included in the analysis because related studies have reported associations between socioeconomic background and health status [46,47]. Table 2 presents definitions of the variables used in the analysis.

## 3. Results

### 3.1. Latent Classes of Health Behavior

According to the latent class model fit criterion, small AIC or adjusted BIC values and large entropy values indicate a favorable model fit. Furthermore, we used an LMR test to compare the improvements in model fit between sequential classes; we considered *p* < 0.0001 to signify a good model fit. On the basis of the model fit indices (Table 3), we identified five latent classes of health behaviors. Table 4 further demonstrates the distribution of health behavior indicators and conditional probabilities in each class. The second column of Table 4 indicates the percentage of the study sample who reported employing each of the health behavior indicators. Overall, 35.1% of respondents reported practicing weight control, 46.4% reported reducing their cigarette smoking or alcohol drinking, 53.7% and 72.3% reported engaging in exercise and following a healthy diet, respectively, and 61.5% and 5.8% reported using relaxation and meditation, respectively, to either maintain or improve their hypertension, diabetes, or hyperlipidemia status.

Columns 3 to 7 present the conditional health behavior probabilities of each indicator identified for the individuals in each class. Conditional probabilities of health behavior indicators are class specific and correspond to the probability that a response is associated with that class. The all-controlled class was classified as Class 1. In this class, the percentage of respondents using each method was relatively high compared with that in other classes. For example, 62.2% of the respondents in this class reported that they used exercise to maintain or improve their hypertension, diabetes, or hyperlipidemia status, whereas 98.3% reported maintaining a healthy diet. Class 2 comprised individuals who had reduced their smoking or drinking habits and employed exercise and relaxation, but not a healthy diet, to control their respective diseases. All (100%) individuals in this class reported using exercise to maintain or improve their hypertension, diabetes, or hyperlipidemia status, whereas 56.1% reported using relaxation methods. However, none (0%) of the respondents reported using a healthy diet to control their diseases. Therefore, this class was designated as the exercise and relaxation class. In Class 3, individuals reported controlling their chronic diseases by reducing smoking or drinking (100%), maintaining a healthy diet (85.2%), and employing relaxation methods (63.8%). We named this class the healthy diet and reduced smoking or drinking class. Individuals in Class 4 reported using a healthy diet (84.8%) to control their chronic diseases. We named this class the healthy diet class. Class 5 was the least-controlled class. In this class, the percentage of respondents who used each method was relatively low compared with that in other classes. Of 2103 individuals, we categorized 32.67%, 9.32%, 15.45%, 26.58%, and 15.98% into Classes 1 to 5, respectively.

### 3.2. Self-Reported Health Status, Analysis of Variance Test, and Pairwise Comparisons for Each Latent Class

We first used an analysis of variance (ANOVA) test to check for a significant difference between the means of self-reported health status among the latent classes. The null hypothesis assumed no significant difference between the means of self-reported health status among the latent classes. The ANOVA test result indicated that the null hypothesis was rejected (F = 11.03, *p* < 0.0001). Therefore, sufficient evidence enabled us to reach the conclusion that not all of the means were equal among the latent classes. To further identify which latent classes were different from one another in terms of self-reported health status, pairwise comparisons were performed. As demonstrated in Table 5, significant comparisons were found between Classes 5 and 1 (0.3082) and Classes 4 and 1 (0.2101). These positive and significant differences indicated that Classes 5 and 4 had poorer self-reported status than Class 1 did. However, no significant differences were found between Classes 3 and 1 (0.0948) and Classes 2 and 1 (0.0892). Although the self-reported health status of Class 1 respondents was still better than that of the respondents in Classes 3 and 2, the differences were nonsignificant. Specifically, individuals in Class 3, that is, the healthy diet and reduced smoking or drinking class, felt that their health status had improved because of their conscious attempt to adopt health behaviors such as reducing smoking or drinking. Additionally, the primary shared health behavior for Classes 2 and 1 was regular exercise. Therefore, it is suggested that regular exercise greatly contributed to the positive perceived health status of Class 2 respondents. Furthermore, we discovered significant and positive differences between the less controlled classes, i.e., Classes 5 and 2 (0.2190) or Class 3 (0.2133). However, no significant differences were found between Classes 5 and 4 (0.0981). For Class 4, which was the healthy diet class, individuals in the class probably needed to engage in more health behaviors than only maintaining a healthy diet. Other nonsignificant comparisons included those between Classes 4 and 2 (0.1209), Classes 4 and 3 (0.1153), and Classes 3 and 2 (0.0056). Different from Class 4, those in Class 2 did not maintain a healthy diet. Compared with Class 3, Class 4 specifically engaged in weight control and health diet methods. Different from Class 2, those in Class 3 attempted to reduce their smoking or drinking levels. Nevertheless, we performed an ordered logit regression for further analysis.

### 3.3. Ordered Logit Model Estimation Results

Table 6 presents the odds ratio (OR) of the five latent classes of health behaviors and other influencing factors associated with self-reported health status estimated by an ordered logit model. The estimation results revealed that individuals in Classes 2, 3, 4, and 5 exhibited poorer health statuses than those in Class 1 (OR = 1.15, 1.29, 1.52, and 2.04, respectively). Therefore, individuals in Classes 2, 3, 4, and 5 tended to have lower levels of self-reported health. However, the result was not significant in Class 2.

Other factors that significantly affected health outcomes were age (respondents aged 70–79 years reported poorer health conditions than those aged 40–49 years, OR = 1.50), education level (those with a college or above education had better outcomes than their counterparts with a junior high school education or below, OR = 0.78), and other disease statuses (those with chronic diseases, such as cancer, heart, stroke, kidney, lung, or liver diseases, or other chronic diseases, such as gout, arthritis, or osteoporosis, had poor health conditions, OR = 1.44 and 1.37, respectively).

Table 7 presents the marginal effects of latent classes of health behavior according to self-reported health status. On average, the probability of Class 2 individuals reporting that their health status was excellent, good, or fair was 0.23, 0.96, and 1.93 percentage points lower than that of those in Class 1, respectively. However, the probability of individuals in Class 2 affirming that their health status was poor or very poor was 2.30 and 0.82 percentage points higher than that of those in Class 1, respectively. Nevertheless, the results for Class 2 were all nonsignificant. On average, the probability of Class 3 individuals affirming that their health status was excellent, good, or fair was 0.43, 1.79, and 3.59 percentage points lower than that of those in Class 1, respectively. However, the probability of Class 3 individuals reporting that their health status was poor or very poor was 4.29 and 1.52 percentage points higher than that of those in Class 1, respectively. On average, the probability of Class 4 individuals reporting that their health status was excellent, good, or fair was 0.71, 2.93, and 5.90 percentage points lower than that of those in Class 1, respectively. However, the probability of individuals in Class 4 indicating that their health status was poor or very poor was 7.04 and 2.49 percentage points higher than that of those in Class 1, respectively. On average, the probability of Class 5 individuals indicating that their health status was excellent, good, or fair was 1.19, 4.95, and 9.95 percentage points lower than that of those in Class 1, respectively. However, the probability of Class 5 individuals indicating that their health status was poor or very poor was 11.88 and 4.21 percentage points higher than that of those in Class 1, respectively.

## 4. Discussion

Studies have highlighted that different health behaviors may co-occur in some individuals but not in others [13,14,15,16,17,18,19,20,21,22,23,24,25,26,27]. Therefore, the use of LCA to evaluate the effects of such heterogeneity on health behavior enabled us to reveal the possible synergistic effects of health behaviors on health status. Our results are consistent with those of related research on healthy lifestyle behaviors. All studies, including ours, have revealed that health behavior classifications obtained using LCA support clear distinctions [13,14,15,16,17,18,19,20,21,22,23,24,25,26,27]. However, the relationship between clusters of health behaviors of individuals with chronic diseases and health status remains understudied. To our knowledge, ours is the first study to investigate the relationship between latent classes of health behaviors and health status by employing LCA for individuals with chronic conditions. Our assessment of the effects of heterogeneity on health behavior indicates the possible synergistic effects on the health status of Taiwanese middle-aged and older adults with hypertension, diabetes, or hyperlipidemia.

The results indicate that, of the five classes, Classes 2, 3, 4, and 5 exhibited poorer health status than those in Class 1—the all-controlled class. However, the result was not significant in Class 2. Specifically, the primary shared method between Classes 1 and 2 that is not present in Class 3 is regular exercise. Relative to Classes 1 and 2, individuals in Class 3 had lower frequencies of engaging in regular exercise. This could explain why Classes 1 and 2 are not clearly separated in terms of health status, whereas Classes 1 and 3 are significantly different in terms of health status. Moreover, Class 3 highlights the health benefits of reducing smoking or drinking. Empirical evidence in the literature has suggested a relationship between healthier patterns of behavior and positive lower levels of psychological distress, higher level of energy vitality, and better self-reported health status and quality of life; having relatively few unhealthy behaviors was associated with less problematic physical and psychosocial health outcomes [13,16]. Our results generalize and extend the corresponding results from previous studies. As for the marginal effects of the latent classes of health behavior for each of the self-reported health statuses, we found a smaller magnitude of coefficient estimates for Class 3 compared with Classes 4 and 5 for individuals who reported their health status as being poor or very poor. This suggests that combining regular exercise, relaxation, and a reduction in smoking or drinking levels leads to better health statuses. For example, the lower self-reported health status of those in Class 4 compared with those in Class 3 indicated that combining several health behaviors beyond controlling diet for Class 4 is critical. Furthermore, the greater magnitude of the coefficient estimates for Class 5, the least-controlled class, compared with Classes 3 and 4 for individuals who reported their health status to be poor or very poor further indicates the importance of preventive medicine.

Although latent classes of health behavior are associated with health status, other variables, namely age, education level, and other chronic disease statuses, are correlated with health status. Compared with individuals aged 40–49 years, those aged 70–79 years and with other chronic diseases reported poorer health conditions. In addition, individuals with a college education or above reported better health conditions than those with a junior high school education or below. Our results indicate that individuals with a higher education level have better health statuses than those with lower education levels, which corresponds to previous studies’ findings [46,47].

The main limitation of this study is that we conducted all analyses on the basis of two items, namely self-reported health status and control of hypertension, diabetes, or hyperlipidemia through lifestyle changes. In this study, individuals’ health behaviors and baseline chronic disease statuses such as hypertension, diabetes, or hyperlipidemia were cross-sectional. A cross-sectional study is an observational study; the responses to each survey question are determined simultaneously for each interviewee. We were unable to determine the exact health status and exact time at which individuals developed hypertension, diabetes, or hyperlipidemia, and the timing and duration of health behavior changes. A second limitation is that we excluded individuals who reported not using tobacco or alcohol. By default, interviewees had to answer the following question from the survey: “Are you currently controlling hypertension, diabetes, or hyperlipidemia through lifestyle changes such as reduction in smoking or drinking?” Therefore, we only included smokers and those who drink alcohol in the analysis. A third limitation is that we used data from the 2005 and 2009 NHIS to conduct the analysis. Health promotion and education campaigns have increased worldwide in recent years. However, studies have highlighted the fact that many individuals do not engage in health behaviors [48,49]. Therefore, our study could provide another perspective on promoting health behaviors using past experiences. Furthermore, applying data from the latest (2013) NHIS is difficult owing to new regulations in Taiwan; the 2013 NHIS is only accessible with restrictions.

## 5. Conclusions

The prevalence of CVD has increased with the widespread prevalence of unhealthy lifestyles. In this study, we addressed the matter of health behaviors by identifying heterogeneous groups of health behaviors among middle-aged and older adults with hypertension, diabetes, or hyperlipidemia in Taiwan. The findings indicate how individuals with chronic diseases can maintain or improve their health status according to their specific health behavior clusters. The results demonstrate that combining several health behaviors rather than a single behavior is critical for maintaining or improving health status. We highlight the importance of health behavior patterns among heterogeneous individuals and its association with their health statuses.

In conclusion, the present study’s results may offer valuable insights for health authorities and medical providers for developing policies and interventions at the tertiary level of the structured model of preventive medicine. We recommend adopting health promotion strategies that encourage engagement in health behaviors specific to each distinct health behavior latent class to maintain or improve the health or chronic disease statuses of middle-aged and older adults. Fleary et al. (2019) reported that clusters of health behaviors have been stable across time among US adults, indicating room for improvement [18]. Health promotion strategies should include not only slogans but also practicable actions. Researchers are recommended to consider conducting a cost-effectiveness analysis of specific health behavior clusters to improve targeted interventions.

## Figures and Tables

**Table 1:** Characteristics of the study sample.

	All
Variables	Proportion (%) or Mean
Self-Reported Health Status	
Excellent	1.71
Good	8.04
Fair	51.69
Poor	32.19
Very Poor	6.37
Personal Characteristics	
Gender/Male	79.17
Married	79.22
HHnumber *	3.1545
Ethnicity	
Taiwanese	71.37
Hakka	12.89
Mainlander	3.23
Other	0.19
Age category	
Age 40–49	29.15
Age 50–59	28.67
Age 60–69	18.88
Age 70–79	16.60
Age 80 or above	6.70
Education Level	
Junior high school or below	58.68
Senior high school	24.39
College or above	16.93
Regional Variables	
Northern area	38.09
Central area	22.49
Southern area	29.24
Eastern area	10.18
Monthly Income Level	
Below NT$40,000	75.32
NT$40,000–NT$79,999	18.78
Above NT$80,000	5.90
Diseases	
Chronic disease (including cancer, heart, stroke, kidney, lung, or liver diseases)	33.10
Other chronic disease (including gout, arthritis, or osteoporosis)	31.76
Mental disease	4.23
Drug Treatment	
Hypertension drug	49.74
Diabetes drug	20.26
Hyperlipidemia drug	17.31
Sample Size	2103

Note: HHnumber * is a continuous variable.

**Table 2:** Definitions of the studied variables.

Variables	Definition
Dependent Variable Self-reported Health Status	self-reported health status compared to that of the previous year: excellent, good, fair, poor, or very poor (from 1 to 5)
Health Behavior Indicator	
Weight Control	if the individual uses weight control to control for hypertension, diabetes, or hyperlipidemia control; yes = 1, else = 0
Smoke or Drink Reduction	if the individual reduces smoke or drink to control for hypertension, diabetes, or hyperlipidemia control; yes = 1, else = 0
Exercise	if the individual uses regular exercise to control for hypertension, diabetes, or hyperlipidemia control; yes = 1, else = 0
Healthy Diet	if the individual uses healthy diet to control for hypertension, diabetes, or hyperlipidemia control; yes = 1, else = 0
Relaxation	if the individual uses relaxation to control for hypertension, diabetes, or hyperlipidemia control; yes = 1, else = 0
Meditation	if the individual use meditation to control for hypertension, diabetes, or hyperlipidemia control; yes = 1, else = 0
Other Control	if the individual uses other methods to control for hypertension, diabetes, or hyperlipidemia control; yes = 1, else = 0
Health Behavior Latent Class	
Class 1: all-controlled	if the individual belongs to this class; yes = 1, else = 0 (the default variable)
Class 2: exercise and relaxation	if the individual belongs to this class; yes = 1, else = 0
Class 3: healthy diet	if the individual belongs to this class; yes = 1, else = 0
Class 4: healthy diet and reduced smoking or drinking	if the individual belongs to this class; yes = 1, else = 0
Class 5: least-controlled	if the individual belongs to this class; yes = 1, else = 0
Personal Characteristics Sex	if the individual’s gender is male; yes = 1, else = 0
Married	if the individual’s marital status; married = 1, else = 0
HHnumber	number of children live with
Ethnicity	
Taiwanese	if the individual is Taiwanese; yes = 1, else = 0
Hakka	if the individual is Hakka; yes = 1, else = 0
Mainlander	if the individual is mainlander; yes = 1, else = 0
Other	if the individual is other ethnicity; yes = 1, else = 0 (the default variable)
Age Category	
Age 40–49	if the individual’s age group is 40–49; yes = 1, else = 0 (the default variable)
Age 50–59	if the individual’s age group is 50–59; yes = 1, else = 0
Age 60–69	if the individual’s age group is 60–69; yes = 1, else = 0
Age 70–79	if the individual’s age group is 70–79; yes = 1, else = 0
Age 80 or above	if the individual’s age group is 80 or above; yes = 1, else = 0
Education Level	
Junior high school or below	if the individual’s education level is illiteracy, elementary school, or junior high school; yes = 1, else = 0 (the default variable)
Senior high school	if the individual’s education level is senior high school; yes = 1, else = 0
College or above	if the individual’s education level is college or above; yes = 1, else = 0
Regional Variables	
Northern area	if the individual is located in Taipei County, Ilan County, Taoyuan County, Hsinchu County, Miaoli County, Taipei City; yes = 1, else = 0
Central area	if the individual is located in Taichung County, Changwa County, Nantou County, Yunlin County, Taichung City; yes = 1, else = 0
Southern area	if the individual is located in Chiayi County, Tainan County, Kaohsiung County, Pingtung County, Kaohsiung City, Chiayi City, Tainan City; yes = 1, else = 0
Eastern area	if the individual is located in Taitung County, Hualien County, Penghu County; yes = 1, else = 0 (the default variable)
Monthly Income Level	
Income 1	if the individual’s monthly income level is below NT$40,000; yes = 1, else = 0 (the default variable)
Income 2	if the individual’s monthly income level is between NT$40,000 and NT$79,999; yes = 1, else = 0
Income 3	if the individual’s monthly income level is NT$80,000 or above; yes = 1, else = 0
Diseases	
Chronic Disease	if the individual has chronic diseases such as cancer, heart, stroke, kidney, lung, or liver diseases; yes = 1, else = 0
Other Chronic Disease	if the individual has gout, arthritis, or osteoporosis; yes = 1, else = 0
Mental	if the individual has mental disease; yes = 1, else = 0
Drug Treatment	
Hypertension drug	if the individual has hypertension drug treatment; yes = 1, else = 0
Diabetes drug	if the individual has diabetes drug treatment; yes = 1, else = 0
Hyperlipidemia drug	if the individual has hyperlipidemia drug treatment; yes = 1, else = 0

**Table 3:** Latent class model fit indices.

	2 Classes	3 Classes	4 Classes	5 Classes	6 Classes
Pearson’s chi-squared	250.423	174.469	125.068	90.822	64.962
LR chi-squared	242.742	160.678	116.547	84.817	70.056
Chi-squared df	112	104	96	88	80
Log-likelihood	−7513.337	−7472.305	−7450.240	−7434.375	−7426.994
AIC	15,056.674	14,990.610	14,962.480	14,946.750	14,947.989
BIC	15,141.441	15,120.586	15,137.665	15,167.143	15,213.592
Adj-BIC	15,093.785	15,047.513	15,039.174	15,043.236	15,064.268
Entropy	0.639	0.551	0.643	0.742	0.734
Vong–Lo–Mendell–Rubin likelihood ratio test (LMR)	1 Versus 2 Classes	2 Versus 3 Classes	3 Versus 4 Classes	4 Versus 5 Classes	5 Versus 6 Classes
LMR p-value	0.0000	0.0000	0.0000	0.0000	0.2069

Note 1: Smaller AIC or adjusted BIC values and larger entropy values indicate a more favorable model fit. Note 2: LMR test compares the improvement in model fit between sequential classes, *p* < 0.0001 is considered to have a good model fit.

**Table 4:** Distribution of health behavior indictors and conditional probabilities per class.

Health Behavior Indicators	Sample Proportion (%)	Class 1: All-Controlled	Class 2: Exercise and Relaxation	Class 3: Healthy Diet and Reduced Smoking or Drinking	Class 4: Healthy Diet	Class 5: Least-Controlled
Weight Control	35.1%	62.2%	14.9%	38.1%	17.9%	2.1%
Smoke or Drink Reduction	46.4%	66.1%	51.2%	100%	0	16.4%
Exercise	53.7%	90%	100%	24.3%	37%	7.4%
Healthy Diet	72.3%	98.3%	0	85.2%	84.8%	0
Relaxation	61.5%	89.6%	56.1%	63.8%	49.3%	12.8%
Meditation	5.8%	13.8%	7.8%	0	0.3%	0.7%
Other Control	9.7%	11.1%	10.6%	4.6%	7.4%	15.7%
Sample Size		687	196	325	559	336
(%)	2103	(32.67%)	(9.32%)	(15.45%)	(26.58%)	(15.98%)

**Table 5:** Pairwise comparisons for each latent class.

Pairwise Comparisons	Difference between the Means of Self-Reported Health Status	*p*-Value
Class 5: least-controlled (mean = 3.5149)—Class 1: all-controlled (mean = 3.2067)	0.3082 ***	0.000
Class 5: least-controlled (mean = 3.5149)—Class 2: exercise and relaxation (mean = 3.2959)	0.2190 **	0.015
Class 5: least-controlled (mean = 3.5149)—Class 3: healthy diet and reduced smoking or drinking (mean = 3.3015)	0.2133 ***	0.004
Class 5: least-controlled (mean = 3.5149)—Class 4: healthy diet (mean = 3.4168)	0.0981	0.356
Class 4: healthy diet (mean = 3.4168)—Class 1: all-controlled (mean = 3.2067)	0.2101 ***	0.000
Class 4: healthy diet (mean = 3.4168)—Class 2: exercise and relaxation (mean = 3.2959)	0.1209	0.330
Class 4: healthy diet (mean = 3.4168)—Class 3: healthy diet and reduced smoking or drinking (mean = 3.3015)	0.1153	0.208
Class 3: healthy diet and reduced smoking or drinking (mean = 3.3015)—Class 1: all-controlled (mean = 3.2067)	0.0948	0.365
Class 3: healthy diet and reduced smoking or drinking (mean = 3.3015)—Class 2: exercise and relaxation (mean = 3.2959)	0.0056	1.000
Class 2: exercise and relaxation (mean = 3.2959)—Class 1: all-controlled (mean = 3.2067)	0.0892	0.615

Note 1: ** significant at the 5% level; *** significant at the 1% level. Note 2: The mean for self-reported health status is 3.3348.

**Table 6:** Ordered logit regression on self-reported status.

Dependent		
Variables: Self-Reported Health Status	**Odds Ratio** **(OR)**	**SE**
Independent Variable Latent Classes		
Class 2	1.1478	0.1805
Class 3	1.2927 *	0.1729
Class 4	1.5247 ***	0.1716
Class 5	2.0369 ***	0.2694
Personal Characteristics		
Gender/Male	0.9955	0.1147
Married	1.0518	0.1184
HHnumber	0.9761	0.0197
Ethnicity		
Taiwanese	0.9692	0.1356
Hakka	1.1940	0.2079
Mainlander	1.0052	0.2974
Age Category		
Age 50–59	1.0170	0.1155
Age 60–69	1.0562	0.1468
Age 70–79	1.5018 ***	0.2267
Age 80 or above	0.9990	0.1991
Education Level		
Senior high school College or above	0.8648 0.7801 *	0.0964 0.1136
Regional Variables		
Northern area	1.2219	0.1999
Central area	1.0652	0.1864
Southern area	1.1606	0.1945
Monthly Income Level		
NT$40,000–NT$79,999	0.8478	0.1042
Above NT$80,000	0.8666	0.1794
Disease		
Chronic disease (including cancer, heart, stroke, kidney, lung, or liver diseases)	1.4377 ***	0.1341
Other chronic disease (including gout, arthritis, or osteoporosis)	1.3674 ***	0.1264
Mental	1.0401	0.2284
Drug Treatment		
Hypertension drug	0.9523	0.0875
Diabetes drug	1.1362	0.1239
Hyperlipidemia drug	1.1099	0.1261
Sample Size	2103	

Note 1: * significant at the 10% level; *** significant at the 1% level. Note 2: Self-reported health status represents excellent, good, fair, poor, or very poor from 1 to 5, respectively. Note 3: The default variable is class 1; age 40–45, the education level is junior high school or below; income level is below NT$40,000; the eastern area. Note 4: The exchange rate for 1 US Dollar to New Taiwan Dollar is approximately 32 in 2006.

**Table 7:** Marginal effect of latent class of health behavior by self-reported health status.

	Excellent	Good	Fair	Poor	Very Poor
	Coefficient (SE)	Coefficient (SE)	Coefficient (SE)	Coefficient (SE)	Coefficient (SE)
Class 2	−0.0023 (0.0027)	−0.0096 (0.0110)	−0.0193 (0.0220)	0.0230 (0.0263)	0.0082 (0.0093)
Class 3	−0.0043 * (0.0024)	−0.0179 * (0.0094)	−0.0359 * (0.0187)	0.0429 * (0.0223)	0.0152 * (0.0080)
Class 4	−0.0071 *** (0.0022)	−0.0293 *** (0.0081)	−0.0590 *** (0.0157)	0.0704 *** (0.0186)	0.0249 *** (0.0069)
Class 5	−0.0119 *** (0.0030)	−0.0495 *** (0.0098)	−0.0995 *** (0.0183)	0.1188 *** (0.0216)	0.0421 *** (0.0085)

Note 1: * significant at the 10% level; *** significant at the 1% level. Note 2: The default variable is class 1.
